# Effects of juvenile hormone in fertility and fertility-signaling in workers of the common wasp *Vespula vulgaris*

**DOI:** 10.1371/journal.pone.0250720

**Published:** 2021-05-17

**Authors:** Cintia Akemi Oi, Helena Mendes Ferreira, Rafael Carvalho da Silva, Andreas Bienstman, Fabio Santos do Nascimento, Tom Wenseleers

**Affiliations:** 1 Laboratory of Socioecology and Social Evolution, KU Leuven, Leuven, Belgium; 2 Departamento de Biologia, Universidade de São Paulo – USP/ Faculdade de Filosofia, Ciências e Letras de Ribeirão Preto, Ribeirão Preto, São Paulo, Brazil; University of Otago, NEW ZEALAND

## Abstract

In the highly eusocial wasp, *Vespula vulgaris*, queens produce honest signals to alert their subordinate workers of their fertility status, and therefore they are reproductively suppressed and help in the colony. The honesty of the queen signals is likely maintained due to hormonal regulation, which affects fertility and fertility cue expression. Here, we tested if hormonal pleiotropy could support the hypothesis that juvenile hormone controls fertility and fertility signaling in workers. In addition, we aimed to check oocyte size as a proxy of fertility. To do that, we treated *V*. *vulgaris* workers with synthetic versions of juvenile hormone (JH) analogue and a JH inhibitor, methoprene and precocene, respectively. We dissected the treated females to check ovary activation and analyzed their chemical profile. Our results showed that juvenile hormone has an influence on the abundance of fertility linked compounds produced by workers, and it also showed to increase oocyte size in workers. Our results corroborate the hypothesis that juvenile hormone controls fertility and fertility signaling in workers, whereby workers are unable to reproduce without alerting other colony members of their fertility. This provides supports the hypothesis that hormonal pleiotropy contributes to keeping the queen fertility signals honest.

## Introduction

Social insect queen pheromones, either produced and released by exocrine glands or present on the cuticle [1–4], are important molecules to be studied because they are key pheromones that regulate eusocial behaviour. There are two main hypotheses which may explain the evolution of queen pheromones: that queens might control the workers to stay in a non-reproductive state, either by actively suppressing reproduction by repressing ovary activation in the workers (queen control hypothesis) [5], or alternatively, queens emit an honest signal about her fertility status, resulting in workers forgoing reproduction (honest signal hypothesis) to respond to their best fitness interests [3].

The honest signal hypothesis finds support from correlational and experimental evidence. For example, van Zweden et al., (2014) [6] demonstrated a correlation between cuticular hydrocarbon (CHC) profiles and fertility in social wasps and also found that reproductive workers will have a CHC profile more similar to CHC profiles of queens than non-reproductive workers. This supports the idea that policing workers would easily discover and attack reproductive individuals [6, 7], suggesting that reproductive individuals are not able to cheat their CHC profile. If only fertile individuals are able to produce this signal, then queen pheromones would be true indices of fertility [8–10]. From experimental evidence, it has been shown that queen pheromones as specific cuticular compounds are conserved across insects that evolved eusociality independently, like bees, wasps, ants and termites [11–16]. A possible explanation for some females being fertile is due to physiologically linked hormones acting in reproduction and production of fertility linked hydrocarbons [1].

Honest signals could also be maintained if they come with a cost, i.e. a handicap, producing these signals is too costly for individuals of lower quality, such as workers, who would be unsuccessful if they would try to take a queens’ place, but only the strongest individual such as a queen, can bear this cost and still be highly reproductive [7, 17]. Looking at the conservation of queen and fertility signals across taxa, it seems that there is no need for a functionating communicative compound to quickly evolve [3, 18]. On the other hand, under the queen control hypothesis, the fast evolution of these fertility signals is expected because there would be an arms race between the queen and the workers [3, 18], which is not observed. There is evidence for the queen mandibular pheromone (QMP) produced by queens of *Apis mellifera* [19, 20] to be a suppressive agent that manipulates the workers against their interests [3, 5, 21], and therefore, supports the queen control hypothesis. The honeybee queen pheromone seems to be a unique and complex system, due to the fact that QMP influences other behaviours (e.g. retinue behaviour or male attraction) and because some studies show that the queen pheromones is a complex of pheromone blends [22]. Although the QMP is an example of queen control hypothesis, recently it was also shown that QMP can also support the honest signal hypothesis, as the most of the QMP compounds were effective to avoid worker reproduction when administered separately in single compounds, indicating honest signal of the queen also a conservation of the QMP as a queen pheromone [23].

For fertility and production of fertility hydrocarbons to be physiologically linked, one hormone, in particular, juvenile hormone (JH), plays a fundamental role [24–27]. In many insect taxa, JH orchestrates metamorphosis, regulates fertility in females, and generates multiple polymorphisms [25, 26, 28]. Specifically, in primitively social insects, the gonadotropic effect of JH has been confirmed in species such as the buff-tailed bumblebee (*Bombus terrestris*) and *Belonogaster* wasps [29, 30], but in *Polistes* it has a context dependent effect [31], affecting species differently and in some cases, JH even regulates aggressive behavior [32, 33]. The pleiotropic effect of JH means that JH affects different processes simultaneously in insects, such as reproduction, immature development, behaviour or physiology [26, 34]. In the highly eusocial wasp, *Vespula vulgaris*, Oliveira et al. (2017) [28] showed that both queen signal production and queen fertility are under shared JH control, showing that hormonal pleiotropy contributes to keep queen signals honest in this species [28].

To investigate the role of juvenile hormone (JH III) in social insects, the synthetic JH analogue (JH) methoprene is often used in research because of its availability and affordability. Previous studies show that topical application or ingestion of methoprene can simulate the effects of pure JH at cellular level [33, 35, 36]. Specifically, application of methoprene as a JH replacement has been done for many insect species, e.g. showing shifts in CHC profile in an African ant (*Myrmicaria eumenoides*) [35], leading to changes in life span and reproduction trade-offs in fruit flies (*Drosophila melanogaster*) [36], inducing aggressive behavior in the European paper wasp (*Polistes dominula*) [33] and fertility and CHC changes in the common wasp (*Vespula vulgaris*) [28]. Opposite to the effects of JH, precocene has been used experimentally in insects as a JH inhibitor, because it destroys the corpora allata and stops the production of JH [37]. Specifically, in the buff-tailed bumblebee (*Bombus terrestris*), precocene has been shown to reduce JH titers, ovary activation, and aggressiveness [38].

Following our previous study, in which we tested the hormonal pleiotropy hypothesis to support the idea that fertility and production of fertility cues are under hormonal control in wasp’s queens [28], in this work, we want to reevaluate the hormonal pleiotropy hypothesis in workers. Because a difference between the treated workers—with methoprene, precocene, and acetone—were not observed previously, with workers mostly showing ovary activation [28], it was suggested that the gonadotropic effect was only present in queens and not the workers. However, recent findings suggest that methoprene can trigger egg laying in workers [39]. Here, we tested if hormonal pleiotropy could support the hypothesis that juvenile hormone controls fertility and fertility signaling in workers. Instead of checking ovary development coded as categories (developed and undeveloped), we investigated oocyte size as a proxy of fertility in *V*. *vulgaris*. Previous literature in Polistinae and Epiponinae wasps used measurements of oocyte size as a proxy of fertility [40–44]. If JH controls fertility through a gonadotropic effect and shifts the fertility signal in workers of *V*. *vulgaris*, this will further support that both are under shared JH control.

## Material and methods

### Experimental setup

Six nests (N = 6 nests) were collected in 2017 in the vicinities of Leuven, Belgium. No permits to collect wasp nests are required. Newly hatched wasps from the six colonies were treated with either 5 μl precocene (15 μg/μl) (Sigma-Aldrich), 5 μl methoprene (20 μg/μl) (Sigma-Aldrich) or 5 μl acetone (VWR chemicals) as control, the chosen concentrations were based on the information available in the literature [28, 39]. From each of the six nests, we divided the total amount of newly emerged workers into four groups (methoprene and acetone control n = 6, precocene and acetone control n = 6), meaning that each nest was a replicate of the experiment (6 nest x 2 experimental boxes—methoprene vs acetone & precocene vs acetone—in total 12 nest boxes). After color marking (Amsterdam, acrylic paint) the wasps on the thorax, one of the treatments was pipetted (Gilson, pipetman) on the abdomen (similar to [28], in which one topical application was demonstrated to be effective). The treated wasps were placed in wooden experimental boxes [28]. Each nest was divided in two, one wooden box was used for precocene treated wasps together with an equal amount of acetone treated wasps, and another wooden box was used for the methoprene treated wasps with an equal amount of acetone treated wasps (S2 Table). Workers treated with hormones were kept in these wooden boxes for 14 days giving the compounds enough time to induce a change in the workers and lead to ovary activation. The wooden boxes had two compartments, one being the nesting room, containing a piece of comb in a dark environment and another being the foraging room containing sugar paste (Apifonda), sugar water (Trim-o-bee extra), a water-saturated cotton *ad libitum* and a daily mealworm. Nest boxes were kept in a climate room with a 12-hour light cycle at 28°C. Depending on the amount of available older workers, 50 to 100 additional older workers were added to the nest boxes to assist the treated workers. After 14 days, the experiment was finished, and all wasps were sedated with CO_2_. The combs were visually inspected for presence of worker-laid eggs, which is characterized by the presence of two or more eggs per cell. Treated wasps were prepared for CHC extraction and dissection.

For chemical analysis, 16 mature queens were additionally sampled in 2017 for chemical comparison. The queens were frozen killed and extracted in 2 ml of pentane for 1 minute. For the treated workers, 1 ml pentane (Acros organics, HPLC) was used for CHC extraction. After 1 minute, wasp bodies were removed, the pentane filled vials were evaporated under the fume hood at room temperature. Glass vials were refilled with 200 μl hexane (HiPerSolv CHROMANORM, HPLC) for workers and 1000 μl hexane for queens. All samples were analyzed using Gas Chromatography-Mass Spectrometry (GC-MS) (Thermo Fisher Scientific Trace 1300 connected to a Thermo Fisher Scientific ISQ mass spectrometer). In the GC, a column of 30 meters with a diameter of 0.25mm and an 0.25 μm film was used (Restek MXT-5). Using splitless injection, 1μl of each sample was injected at 320°C. Initially, the temperature was held at 40°C for 2 min, then increased to 120°C with an increase of 20°C min^-1^. Followed by an increase of 10°C min^-1^ until 200°C, then 7°C min^-1^ until 250°C and the last increase of 5°C to 350°C min^-1^ which was held for 4 minutes. The carrier gas helium had a constant flow rate of 0.9 ml min^-1^. For the MS electric ionization was used at 300°C which was also the temperature of the MS transfer line. The MS scan time started with a 5-minute delay in a range of 33–720 amu to avoid hexane pollution in the chromatograms. To identify the different hydrocarbons and calculate retention indexes, alkane ladders (Supelco) were run and used in the range of *n*-heptane (C_7_) to *n*-tetracontane (C_40_) to calculate the retention indexes. Ion chromatograms were integrated using R studio (R version 3.6) with a script previously developed in the lab (Supplemental Material). Amdis (version 2.71) was used to identify compounds using known ion fragmentation of cuticular hydrocarbons.

### Analysis of ovary activation

**Fig 1:**
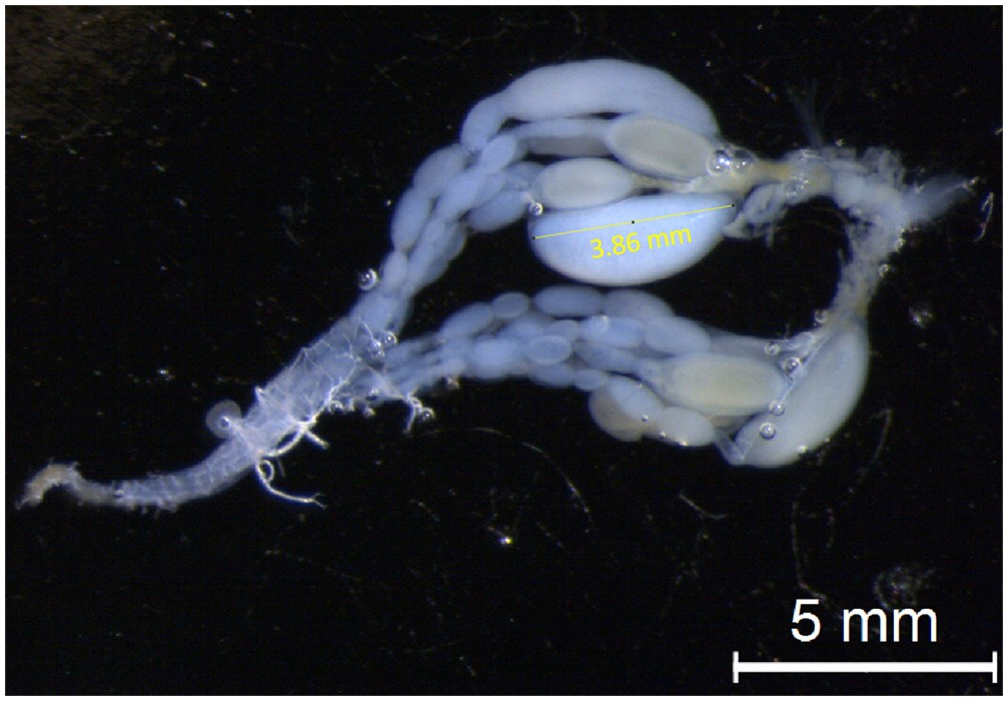
Measurement of oocyte size in *Vespula vulgaris* workers. The yellow bar indicates the oocyte length measure with ImageJ.

Dissection was performed in a ringer solution to create an isotonic solution preventing swelling or shrinking of the tissues. Pictures of the oocytes were taken using a stereomicroscope (Leica, model: MSV266) with a camera linked to a computer using the Leica Application Suite program. Using visual assessment, the biggest oocytes were measured in mm using ImageJ (v.1.58j8) (Fig 1). As we are using a size measure for fertility, we included head size in the analysis to account for the size of each individual. After decapitating and removing the antennae, each head was placed under the stereomicroscope to achieve a frontal view. The head size was measured on the widest part of the face, using the same hardware and software as for the ovary measurements.

For statistical analysis, ovary activation between treated and control individuals was analyzed using a linear mixed model, fitted by the *lmer* function [45] in the *afex* package [46]. The model was fitted with the log-transformed biggest oocyte size as a fixed effect, including head size per wasp as covariate and nest of origin along with the specific wooden box as random factors. Anova was performed and pairwise comparisons were obtained by Tukey posthoc tests with the *contrast* and *lsmeans* functions from the *lsmeans* package [45].

### Analysis of CHC profiles

Differences in compound abundancy between treatments were analyzed by an ANOVA on log-transformed relative peak areas per compound. Using the functions *contrast* and *lsmeans* Tukey posthoc tests (package *lsmeans* [45]) with Bonferroni corrections were performed to compare the abundance of each compound between treatments. The function *pheatmap* from the *pheatmap* package [47] was used to visualize the data with UPGMA as a clustering method using 1-Pearson correlation for the distance matrix.

## Results

### Ovary activation

**Fig 2:**
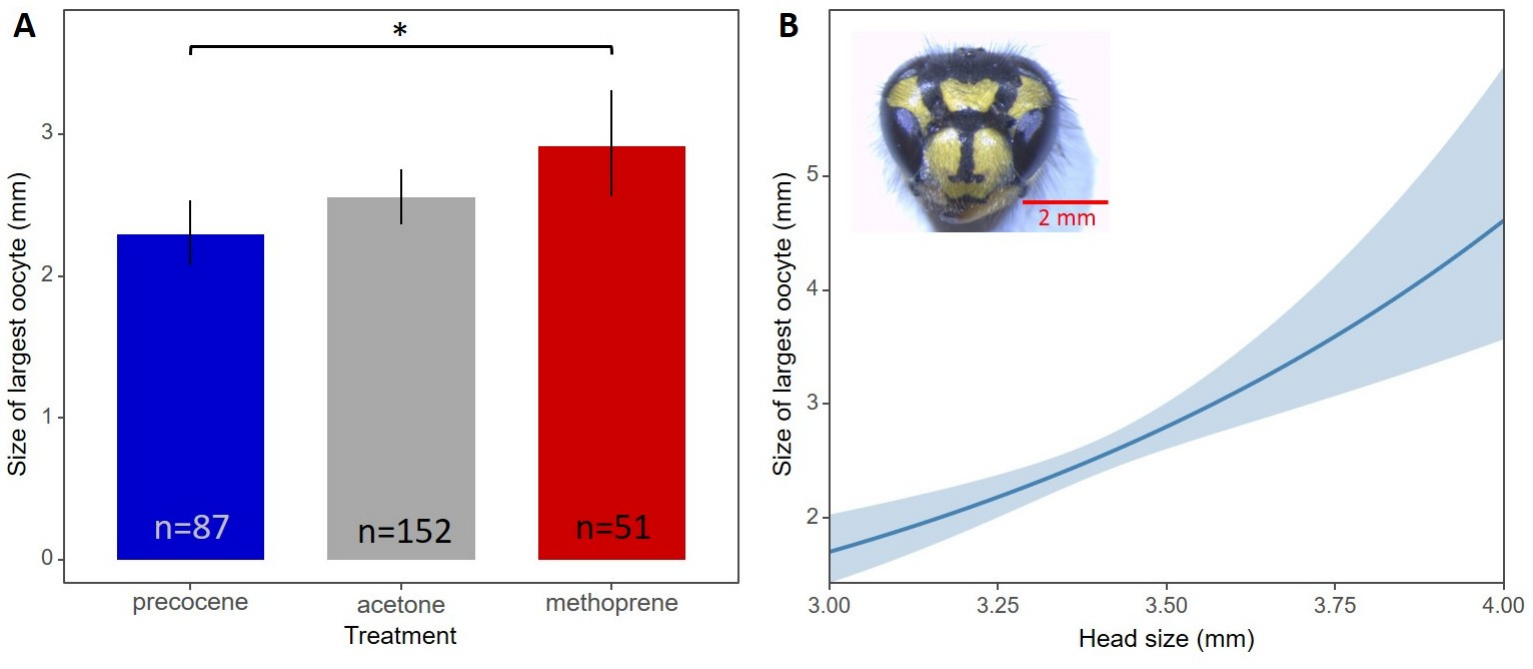
(A) Oocyte size is largest in methoprene treatment, followed by acetone and precocene. 1-tailed pairwise contrasts shows a significant difference between all treatments size. The control treatment acetone did not differ from both the methoprene (t ratio = -1.76, p = 0.081) and precocene (t ratio = 1.68, p = 0.097) treatments, but between methoprene and precocene treatments the biggest oocyte size differs significantly (t ratio = 2.64, p = 0.013). (B) Effect plot of model fitted based on the measurements of the head width of workers.

In total we dissected 290 workers of *V*. *vulgaris*. The topical application of JH showed a gonadotropic effect in workers when compared oocyte size between methoprene and precocene treated workers. We compared ovary activation between treatments, showing significant differences in biggest oocyte size (χ^2^ = 8.68, *p* = 0.013) and 2-tailed pairwise contrasts show a significant difference between methoprene and precocene (Table 1, Fig 2). The control treatment (acetone) does not significantly differ from either the methoprene (*t ratio* = -1.75, *p* = 0.081) or precocene (*t ratio* = 1.67, *p* = 0.097) treatments. Between methoprene and precocene treatments the biggest oocyte size does differ significantly (*t ratio* = 2.64, *p* = 0.013). Oocyte size differences between precocene treated workers and acetone treated workers, and methoprene treated workers and acetone treated were similar but in opposite directions: methoprene increased oocyte size and precocene decreased oocyte size. An important note is the influence of the wasp body size, represented by the head size on the biggest oocyte size. There is an increase in oocyte size correlated with head sizes. The linear mixed model takes this into account and shows a significant effect of head size on the biggest oocyte size (χ^2^ = 22.35, *p* = 0.000). The difference of the number of individuals analyzed between the treatments (S2 Table) are due to the mortality that occurred during the experiment, with higher mortality in methoprene treated individuals (S3 Table).

**Table 1:** Oocyte size across hormonal treatments in *Vespula vulgaris* workers compared using a general linear mixed model (GLMM) with treatment and head size included as fixed effects and nest and nestbox coded as random factors.

**A) Random effects**					
**Groups**	**estimate**	**SE**			
nest box	0.00	0.02			
Nest	0.00	0.00			
Residual	0.03	0.19			
**B) Fixed effects**					
	**(Intr)**	**methoprene**	**precocene**		
**Methoprene**	-0.33				
**Precocene**	0.31	-0.53			
**head size**	0.02	-0.05	0.01		
**C) Anova**					
	**Chisquare**	**Df**	**pr(>Chisq)**		
**(intercept)**	855.05	1.00	0.000	***	
**treatment**	8.68	2.00	0.013	*	
**head size**	22.35	1.00	0.000	***	
**D) Pairwise comparisons**					
**Contrast**	**estimate**	**SE**	**Df**	**t ratio**	***p value***
**acetone-methoprene**	-0.06	0.03	155.60	-1.76	0.081
**acetone-precocene**	0.05	0.03	97.60	1.68	0.097
**methoprene-precocene**	0.10	0.04	29.60	2.64	0.013

(A) Random effects; (B) Fixed effects. (C) Anova; (D) Tukey post-hoc comparisons were performed to determine significant effects of treatment on oocyte size. Coefficients, standard error (SE), t ratio, degrees of freedom (Df) and p-values of the random effect intercepts are shown. Significance codes: p < 0.001 ***, p < 0.01 **, p < 0.05 *.

### CHC analysis

In the chemical analysis of the cuticle, we identified a total of 68 hydrocarbons (S1 Table), consisting of linear alkanes, alkenes, methylated and di-methylated alkanes.

**Fig 3:**
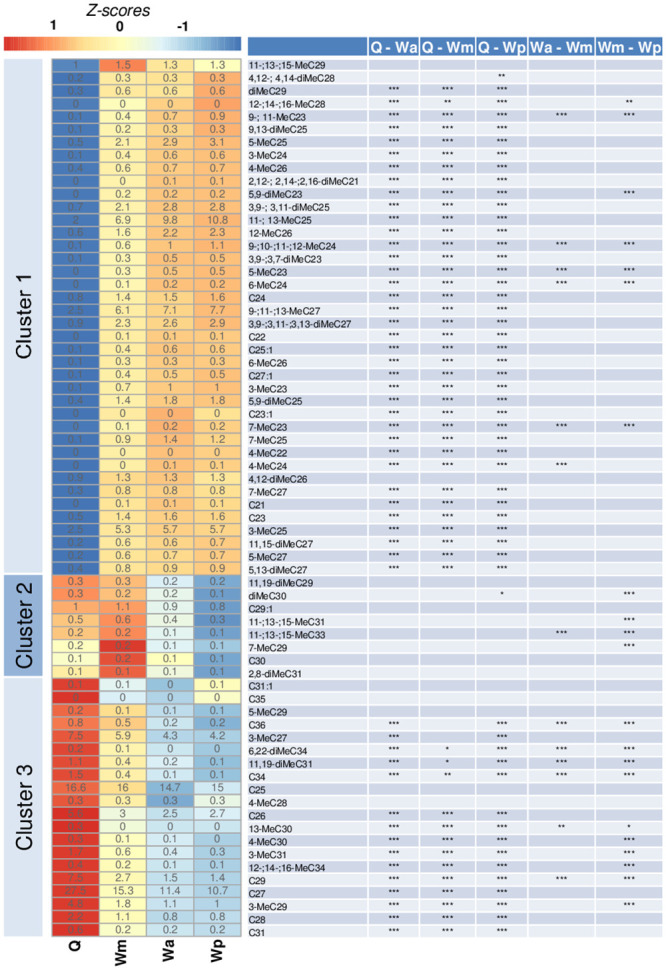
On the left, a heatmap based on the differences in cuticular hydrocarbon profiles on treated individuals, on the right showing all the identified compounds with their significance contrasts based on p-values. Queens are pooled (Q) and different worker treatments (Wm, Wa and Wp) represent methoprene, acetone and precocene, respectively. Contrasts were not significant between Wa-Wp, therefore are not shown. The numbers in the heatmap shows the average relative peak area per compound for each treatment, the background color represents the z-scores. Clusters were obtained by UPGMA clustering and 1-Pearson correlation for the distance matrix. Tukey posthoc contrasts between treatments are obtained from a linear mixed model with Bonferonni correction: *p < 0.05, **p < 0.01, ***p < 0.001.

The chemical comparison between the treated groups (workers treated with methoprene—Wm; workers treated with precocene—Wp; workers treated with acetone—Wa and queens—Q) exhibited significant differences for multiple compounds (Fig 3). No difference between workers treated with acetone (Wa) and workers treated with precocene (Wp) was found, therefore, we excluded this column from the heatmap. The heatmap UPGMA cluster analysis shows the average relative peak areas groups for the compounds in three clusters (Fig 3). Cluster 1 includes compounds characteristic of workers, where significant differences in compound abundancy are mainly between workers and queens. In this cluster, we can find compounds that are more specific to workers than queens, for example, the compounds 7-MeC23, 5,9-diMeC23, and 6-MeC24. These compounds differed significantly between methoprene and precocene treated workers (Fig 3). In the cluster 2, it is shown the compounds that are upregulated with methoprene treatment and downregulated with precocene treatment and therefore showed a significant difference in abundancies both in methoprene and precocene treated workers. Cluster 3 contains the queen pheromones n-C27, n-C28, n-C29, and 3-MeC29 along with other compounds that have high relative peak areas and high z-scores, indicating upregulation of these pheromones in queens. Differences in relative compound abundancy in these queen pheromones were found between queens and workers and between different worker treatments for n-C29 and 3-MeC29. Another compound with high relative abundance in queens compared to workers is 3-MeC27 with an average relative peak area of 7.5 for queens and 5.9, 4.3, and 4.2 for methoprene, acetone, and precocene treated workers, respectively. The linear alkane n-C26 has a higher abundance in queens compared to workers, with an average relative peak area of 5.6, compared to an average relative peak area of 3.0, 2.5, and 2.7 for methoprene, acetone, and precocene treated workers, respectively. n-C26 only shows significant differences in average relative peak area between queens and workers (all three groups), 3-MeC27 average relative peak areas seemed not to differ significantly between methoprene treated workers and queens. n-C25 has a high average relative peak area of 16.6 in queens, but does not seem to be strongly upregulated, for example, the lowest average relative peak area is 14.7 for acetone treated workers.

Furthermore, we saw a trend for shorter-chain saturated alkanes to be present in cluster 1, containing n-C21 to n-C24 (Fig 3). Longer-chain saturated alkanes, apart from n-C30, are present in cluster 2, and the other long-chain alkanes such as n-C25 to n-C31 and n-C34 to n-C36 all appeared in the cluster 3. For methylated compounds, we saw a similar result, with cluster 1 containing methylated alkanes with a backbone of 21 to 29 carbons. Cluster 2 and cluster 3 only showed methylated alkanes with a backbone chain length of 28 carbons or higher, up to 33 carbons for cluster 2, and 34 carbons for cluster 3.

All 68 compounds are both present in queens and workers but differ in abundance between worker treatments and castes. In total, 21 compounds significantly differed in abundance between methoprene and precocene workers and 13 significantly differed in abundance between methoprene workers and the control group. Of all 68 compounds, 51 significantly differed in abundancy between queens and methoprene workers. Comparing the queens with the different worker treatments, 4 extra compounds, besides the other 51 compounds, were significantly different between queens and precocene treated workers.

## Discussion

Our results provide evidence that JH influences both fertility and fertility signaling in workers, as both are under shared hormonal control in *V*. *vulgaris*, and supports the hormonal pleiotropy hypothesis [28]. The results of oocyte size combined with the chemical analyses imply that fecund workers, who usually remain sterile, are unable to secretly cheat their chemical profile and fertility signal, which probably could contribute to alert other workers that can police them [39, 48–50]. This assumption is yet to be investigated in *V*. *vulgaris*, or other Vespine wasps. It is suggested that workers attempting to cheat in queenright colonies may be constrained by simultaneous aggression and egg removal policing: if reproductive workers produce fertility cues for their eggs to avoid egg removal, then perhaps reproductive workers are unable to avoid expressing them (fertility cues) on their cuticles and receiving aggressive responses from other colony mates [51].

Even though there was no statistical significance in oocyte size between the acetone control and methoprene treatment, methoprene treated workers tended to express bigger oocytes when compared with control workers. In addition, methoprene treated workers had higher similarities of CHC profiles compared with the queens. Some of the queen fertility compounds in the methoprene treated workers showed higher abundances compared to the controls, supporting the idea of fertility linked cues. Comparing these results with previous data from Oliveira et al. (2017) [28], we reached different conclusions on the fertility of the workers, probably because we used higher precocene dosages (15μg/μl vs. 6μg/μl), which did not noticeably affect the mortality rate. Additionally, we included the analysis of oocyte size, instead of categorizing ovary activation, whereas Oliveira et al. (2017) found mostly workers to have activated ovaries. As initially hypothesized, our results provide experimental evidence of a gonadotropic effect of JH in workers of *V*. *vulgaris*.

Additionally, no significant difference between the control oocyte size was found across those workers treated with methoprene or precocene, because the comparison of worker-like states is smaller, and so, those oocyte differences are only possible to be seen checking opposite effects of methoprene and precocene. Although our results seem to be less robust compared to that previous study [28], our findings are not less of importance, and need to be taken together with previous literature. The gonadotropic effect of JH was clear for queens of *V*. *vulgaris* [28], now it is more evident for workers for several reasons, workers in queenless situations treated with JH activate their ovaries [28], changes their CHC ([28] and this work), anticipates egg-laying [39], show larger oocytes (this work) and changes the chemistry of their eggs [39].

Furthermore, our findings are in agreement with other studies using different insect species in which they found a gonadotropic effect of JH, for example, in *V*. *vulgaris*, anticipating ovary activation [39], in *Polistes* wasps (*Polistes metricus* and *Polistes dominula*) [31, 52], in bumblebees (*Bombus terrestris*) [29] and Epiponini wasp *Polybia micans* [53]. Comparing highly eusocial species, with big colony sizes and highly eusocial behavior, as honeybees, some termites and ants, the gonadotropic effect of JH is absent, but still plays an import role in honeybees, where JH titers correlate with age-related division of labor [25, 27]. JH is one of the main hormones in insects and it seems that its effects change with increasing levels of sociality, where it still has a gonadotropic effect in solitary, primitively eusocial, and highly social species with modest colony sizes, but loses this gonadotropic effect in highly eusocial species with large colony sizes [27], although no conclusive function is found especially for primitively eusocial species [27].

The methodology of a single topical application of the synthetic version of the hormones may not be ideal since the concentration of methoprene and precocene are assumed to degrade over time. Assuming a decay in methoprene and precocene concentration over time, we do not know how long the applied amount would be effectively absorbed through the cuticle, and how long it would be circulating in the hemolymph to demonstrate any effects. For example, Wilson et al. (1983) [54] showed that the corpora allata can recover after 48h of applying precocene in *Drosophila melanogaster*, indicating that multiple applications may be necessary. Further research investigating the neurological effects of precocene could elucidate this question by determining the ideal precocene concentrations to successfully suppress the corpora allata in a non-lethal manner. Considering these toxic effects of both compounds used in our experiments, only the dosages of methoprene influenced survivability.

We found a positive correlation between oocyte size and body size (head width as a proxy) in *V*. *vulgaris*. In workers of *V*. *vulgaris*, it is possible that bigger workers can invest more in their own reproduction instead of the smaller workers, since they may have a bigger lipid reserve. There is few evidences that there is a correlation between body size and ovary activation in workers of social insects, for example, it has been shown for the solitary bee *Megachile rotundata*, that females that lay bigger eggs also have a larger head size [55]. In *Bombus impatiens*, there was also a correlation in head width and oocyte size in workers of a queenless colony [56]. However, in some *Polistes* species, body size is a significant factor explaining a wasp’s rank, where the highest-ranked wasp reproduces [57]. Future studies in reproductive workers in other social insect species are needed to understand how size may influence fertility [56, 57].

Within the chemical profiles of both *V*. *vulgaris* queens and workers, we identified the known queen pheromones such as heptacosane (n-C27), octacosane (n-C28), nonacosane (n-C29), and 3-methylnonacosane (3-MeC29), which were more abundant in queens than in workers, similar to the previous literature [11, 28]. For methoprene treated workers, we showed an upregulation in these queen pheromones but also, for hexacosane (n-C26) and 3-methylheptacosane (3-MeC27). Overall, methoprene treated workers seem to be more similar towards queen profiles which provides evidence that JH shapes the chemical profile of workers. This is in agreement with studies searching the effect of JH on CHC profile in ants, where they suggest that an increase in JH increases the abundance of queen characteristic hydrocarbons [58]. In contrast, precocene treated workers showed no difference from acetone controls. This could be due to either the concentration of precocene applied not being ideal or that the destruction of corpora allata caused by precocene in the adult wasp would not change the CHC composition within the two weeks of experiment. Chemical profiles of workers appear to have hydrocarbons with shorter chain length (between heneicosane n-C21 to tetracosane n-C24 and up to nonacosane n-C29 for methylated hydrocarbons), where queens exhibit in their chemical profile longer chained hydrocarbons (from pentacosane n-C25 to heintriacontane n-C31 and from octacosane n-C28 to tetratriacontane n-C34 for methylated hydrocarbons). These results are in agreement with the literature in vespine wasps, where they identified hydrocarbons of longer chain length to be more abundant on the queens and reproductive workers cuticles, suggesting that these longer-chained compounds are linked to fertility [6, 59].

In conclusion, our results show that JH affects ovary activation levels in workers of *V*. *vulgaris* and support the hormonal pleiotropy hypothesis in which the fertility and production of fertility cues are under shared endocrine control.

## Supporting information

S1 Data (RAR)

S1 Table Identification of 68 compounds in the cuticle of workers (n = 308) and queens (n = 16). Retention time (min), Kovats retention indices and diagnostic ions per compound.
(DOCX)

S2 Table Initial number of treated wasps and total number of treated alive and dead wasps during our experiment (NA: Not available). (DOCX)

S3 Table Pairwise comparison of the proportion of individuals that died in the treatments, showing that methoprene treated individuals were different from acetone control and precocene. Binomial GLMM, with colony and nest box as random intercept. To check significant differences between treatments were calculated using Tukey post-hoc test (function lsmeans of the emmeans package). Odds-ratio, standard errors, z-ratio and p-values are shown.
(DOCX)

## References

[1] Oi CA, van Zweden JS, Oliveira RC, Van Oystaeyen A, Nascimento FS, Wenseleers T. The origin and evolution of social insect queen pheromones: Novel hypotheses and outstanding problems. Bioessays. 2015;37:808–21. 10.1002/bies.201400180 25916998

[2] Billen J, Morgan ED. Pheromone communication in social insects: sources and secretions. In: Vander Meer RK, Breed MD, Espelie KE, Winston ML, editors. Pheromone Communication in Social Insects—Ants, Wasps, Bees, and Termites. Boulder, Colorado: Westview Press; 1998. p. 3–33.

[3] Keller L, Nonacs P. The role of queen pheromones in social insects: queen control or queen signal? Anim Behav. 1993;45(4):787–94. 10.1006/anbe.1993.1092

[4] Kocher SD, Grozinger CM. Cooperation, conflict, and the evolution of queen pheromones. J Chem Ecol. 2011;37:1263–75. 10.1007/s10886-011-0036-z 22083225

[5] Fletcher DJC, Ross KG. Regulation of Reproduction in Eusocial Hymenoptera. Annu Rev Entomol. 1985;30(1):319–43. 10.1146/annurev.en.30.010185.001535

[6] van Zweden JS, Bonckaert W, Wenseleers T, d’Ettorre P. Queen signalling in social wasps. Evolution. 2014;68(4):976–86. 10.1111/evo.12314 24219699

[7] van Zweden JS. The evolution of honest queen pheromones in insect societies. Commun Integr Biol. 2010;3(1):50–2. 10.4161/cib.3.1.9655 20539783 PMC2881241

[8] Holman L. Queen pheromones: the chemical crown governing insect social life. Commun Integr Biol. 2010;3:558–60. 10.4161/cib.3.6.12976 21331238 PMC3038062

[9] Holman L. Queen pheromones and reproductive division of labor: a meta-analysis. Behav Ecol. 2018;29(6):1199–209. 10.1093/beheco/ary023

[10] Peso M, Elgar MA, Barron AB. Pheromonal control: reconciling physiological mechanism with signalling theory. Biol Rev. 2015;90(2):542–59. 10.1111/brv.12123 24925630

[11] Van Oystaeyen A, Oliveira RC, Holman L, Van Zweden JS, Romero C, Oi CA, et al. Conserved class of queen pheromones stops social insect workers from reproducing. Science. 2014;287:287–90. 10.1126/science.1244899 24436417

[12] Funaro CF, Böröczky K, Vargo EL, Schal C. Identification of a queen and king recognition pheromone in the subterranean termite *Reticulitermes flavipes*. Proc Natl Acad Sci USA. 2018. 10.1073/pnas.1721419115 29555778 PMC5899469

[13] Holman L, Hanley B, Millar JG. Highly specific responses to queen pheromone in three *Lasius* ant species. Behav Ecol Sociobiol. 2016;70(3):387–92. 10.1007/s00265-016-2058-6

[14] Holman L, Jørgensen CG, Nielsen J, d’Ettorre P. Identification of an ant queen pheromone regulating worker sterility. Proc R Soc Lond B Biol Sci. 2010;277:3793–800. 10.1098/rspb.2010.0984 20591861 PMC2992706

[15] Holman L, Lanfear R, d’Ettorre P. The evolution of queen pheromones in the ant genus *Lasius*. J Evol Biol. 2013;26(7):1549–58. 10.1111/jeb.12162 23662630

[16] Nunes TM, Oldroyd BP, Elias LG, Mateus S, Turatti IC, Lopes NP. Evolution of queen cuticular hydrocarbons and worker reproduction in stingless bees. Nat Ecol Evol. 2017;1:0185. 10.1038/s41559-017-0185

[17] Zahavi A. Mate selection—A selection for a handicap. J Theor Biol. 1975;53(1):205–14. 10.1016/0022-5193(75)90111-3 1195756

[18] Heinze J, d’Ettorre P. Honest and dishonest communication in social Hymenoptera. J Exp Biol. 2009;212(12):1775–9. 10.1242/jeb.015008 19482994

[19] Hoover SER, Keeling CI, Winston ML, Slessor KN. The effect of queen pheromones on worker honey bee ovary development. Naturwissenschaften. 2003;90(10):477–80. 10.1007/s00114-003-0462-z 14564409

[20] Slessor KN, Winston ML, Le Conte Y. Pheromone communication in the honeybee (*Apis mellifera* L.). J Chem Ecol. 2005;31:2731–45. 10.1007/s10886-005-7623-9 16273438

[21] Strauss K, Scharpenberg H, Crewe RM, Glahn F, Foth H, Moritz RFA. The role of the queen mandibular gland pheromone in honeybees (*Apis mellifera*): honest signal or suppressive agent? Behav Ecol Sociobiol. 2008;62(9):1523–31. 10.1007/s00265-008-0581-9

[22] Hefetz A, Katzav-Gozansky T. Are multiple honeybee queen pheromones indicators for a queen-workers arms race. Apiacta. 2004;39:44–52.

[23] Princen SA, Oliveira RC, Ernst U, Millar JG, van Zweden JS, Wenseleers T. Honeybees possess a structurally diverse and functionally redundant set of queen pheromones. Proc R Soc Lond B Biol Sci. 2019;286(1905):20190517. 10.1098/rspb.2019.0517 31213188 PMC6599994

[24] Santos CG, Humann FC, Hartfelder K. Juvenile hormone signaling in insect oogenesis. Curr Opin Ins Sci. 2019;31:43–8. 10.1016/j.cois.2018.07.010 31109672

[25] Hartfelder K, Emlen D. Endocrine control of insect polyphenism. In: L.I G, editor. Insect Endocrinology. San Diego, CA: Elsevier; 2012. p. 464–522.

[26] Hartfelder K. Insect juvenile hormone: from "status quo" to high society. Braz J Med Biol Res. 2000;33(2):157–77. 10.1590/s0100-879x2000000200003 10657056

[27] Huang ZY. Juvenile Hormone. In: Starr CK, editor. Encyclopedia of Social Insects. Cham: Springer International Publishing; 2020. p. 1–3.

[28] Oliveira RC, Vollet-Neto A, Akemi Oi C, van Zweden JS, Nascimento F, Sullivan Brent C, et al. Hormonal pleiotropy helps maintain queen signal honesty in a highly eusocial wasp. Sci Rep. 2017;7(1):1654. 10.1038/s41598-017-01794-1 28490760 PMC5431770

[29] Shpigler H, Amsalem E, Huang ZY, Cohen M, Siegel AJ, Hefetz A, et al. Gonadotropic and physiological functions of juvenile hormone in bumblebee (*Bombus terrestris*) workers. PloS one. 2014;9(6):e100650. 10.1371/journal.pone.0100650 24959888 PMC4069101

[30] Kelstrup HC, Hartfelder K, Esterhuizen N, Wossler TC. Juvenile hormone titers, ovarian status and epicuticular hydrocarbons in gynes and workers of the paper wasp *Belonogaster longitarsus*. J Insect Physiol. 2017;98:83–92. 10.1016/j.jinsphys.2016.11.014 27913150

[31] Kelstrup HC, Hartfelder K, Wossler TC. *Polistes smithii* vs. *Polistes dominula*: the contrasting endocrinology and epicuticular signaling of sympatric paper wasps in the field. Behav Ecol Sociobiol. 2015;69(12):2043–58. 10.1007/s00265-015-2015-9

[32] Tibbetts EA, Izzo AS. Endocrine mediated phenotypic plasticity: condition-dependent effects of juvenile hormone on dominance and fertility of wasp queens. Horm Behav. 2009;56(5):527–31. 10.1016/j.yhbeh.2009.09.003 19751736

[33] Tibbetts EA, Fearon ML, Wong E, Huang ZY, Tinghitella RM. Rapid juvenile hormone downregulation in subordinate wasp queens facilitates stable cooperation. Proc R Soc Lond B Biol Sci. 2018;285(1872). 10.1098/rspb.2017.2645 29436498 PMC5829203

[34] Jindra M, Bellés X, Shinoda T. Molecular basis of juvenile hormone signaling. Curr Opin Ins Sci. 2015;11:39–46. 10.1016/j.cois.2015.08.004 28285758

[35] Lengyel F, Westerlund S, Kaib M. Juvenile hormone III influences task-specific cuticular hydrocarbon profile changes in the ant *Myrmicaria eumenoides*. J Chem Ecol. 2007;33(1):167–81. 10.1007/s10886-006-9185-x 17146723

[36] Flatt T, Tu M-P, Tatar M. Hormonal pleiotropy and the juvenile hormone regulation of *Drosophila* development and life history. Bioessays. 2005;27(10):999–1010. 10.1002/bies.20290 16163709

[37] Bowers WS. How anti-juvenile hormones work. Am Zool. 1981;21(3):737–42. 10.1093/icb/21.3.737

[38] Amsalem E, Teal P, Grozinger CM, Hefetz A. Precocene-I inhibits juvenile hormone biosynthesis, ovarian activation, aggression and alters sterility signal production in bumble bee (*Bombus terrestris*) workers. The Journal of Experimental Biology. 2014;217(17):3178–85. 10.1242/jeb.107250 25013106

[39] Oi CA, Brown RL, Da Silva RC, Wenseleers T. Reproduction and signals regulating worker policing under identical hormonal control in social wasps. Sci Rep. 2020;10(18971). 10.1038/s41598-020-76084-4 33149171 PMC7643062

[40] Giray T, Giovanetti M, West-Eberhard MJ. Juvenile hormone, reproduction, and worker behavior in the neotropical social wasp *Polistes canadensis*. Proc Natl Acad Sci USA. 2005;102(9):3330–5. 10.1073/pnas.0409560102 15728373 PMC552932

[41] Tibbetts EA, Sheehan MJ. The effect of juvenile hormone on *Polistes* wasp fertility varies with cooperative behavior. Horm Behav. 2012;61(4):559–64. 10.1016/j.yhbeh.2012.02.002 22349082

[42] Kelstrup HC, Hartfelder K, Nascimento FS, Riddiford LM. The role of juvenile hormone in dominance behavior, reproduction and cuticular pheromone signaling in the caste-flexible epiponine wasp, *Synoeca surinama*. Front Zool. 2014;11(1):1–19. 25371699 10.1186/s12983-014-0078-5 PMC4219083

[43] da Silva RC, Prato A, Oi CA, Turatti ICC, Nascimento FS. Dominance Hierarchy, ovarian activity and cuticular hydrocrabons in the primitively eusocial wasp *Mischocyttarus cerberus* (Vespidae, Polistinae, Mischocyttarini). J Chem Ecol. 2020. 10.1007/s10886-020-01206-1 32789711

[44] Walton A, Tumulty JP, Toth AL, Sheehan MJ. Hormonal modulation of reproduction in *Polistes fuscatus* social wasps: Dual functions in both ovary development and sexual receptivity. J Insect Physiol. 2020;120:103972. 10.1016/j.jinsphys.2019.103972 31705844 PMC7558460

[45] Bates D, Mächler M, Bolker B, Walker S. Fitting Linear Mixed-Effects Models Using lme4. J Stat Soft. 2015;67(1). 10.18637/jss.v067.i01

[46] Singmann H, Bolker B, Westfall J, Aust F. afex: Analysis of Factorial Experiments. R package version 0.16 https://CRAN.R-project.org/package=afex ed2016.

[47] Kolde R. pheatmap: Pretty heatmaps. R package https://cran.r-project.org/web/packages/pheatmap ed2015.

[48] Ratnieks FLW. Reproductive harmony via mutual policing by workers in eusocial Hymenoptera. Am Nat. 1988;132:217–36. 10.1086/284846

[49] Wenseleers T, Hart A, Ratnieks F. When resistance is useless: Policing and the evolution of reproductive acquiescence in insect societies. Am Nat. 2004;164:E154–E67. 10.1086/425223 29641925

[50] Ratnieks FLW, Foster KR, Wenseleers T. Conflict resolution in insect societies. Annu Rev Entomol. 2006;51:581–608. 10.1146/annurev.ento.51.110104.151003 16332224

[51] Smith AA, Hölldober B, Liebig J. Cuticular hydrocarbons reliably identify cheaters and allow enforcement of altruism in a social insect. Curr Biol. 2009;19(1):78–81. 10.1016/j.cub.2008.11.059 19135369

[52] Bohm MK. Effects of environment and juvenile hormone on ovaries of the wasp, *Polistes metricus*. J Insect Physiol. 1972;18(10):1875–83. 10.1016/0022-1910(72)90158-8

[53] Kelstrup HC, Hartfelder K, Nascimento FS, Riddiford LM. Reproductive status, endocrine physiology and chemical signaling in the Neotropical, swarm-founding eusocial wasp *Polybia micans*. J Exp Biol. 2014;217(13):2399–410. 10.1242/jeb.096750 24744417 PMC4081010

[54] Wilson TG, Landers MH, Happ GM. Precocene I and II inhibition of vitellogenic oöcyte development in *Drosophila melanogaster*. J Insect Physiol. 1983;29(3):249–54. 10.1016/0022-1910(83)90091-4

[55] O’Neill KM, Delphia CM, O’Neill RP. Oocyte size, egg index, and body lipid content in relation to body size in the solitary bee *Megachile rotundata*. PeerJ. 2014;2:e314–e. 10.7717/peerj.314 24711966 PMC3970799

[56] Melgarejo V, Wilson Rankin EE, Loope KJ. Do queen cuticular hydrocarbons inhibit worker reproduction in *Bombus impatiens*? Insectes Soc. 2018;65(4):601–8. 10.1007/s00040-018-0651-6

[57] Reeve H. Polistes. In: Ross K, Matthews R, editors. The social biology of wasps. Ithaca, NY: Cornell University Press; 1991.

[58] Holman L. Costs and constraints conspire to produce honest signalling: Insights from an ant queen pheromone. Evolution. 2012;66(7):2094–105. 10.1111/j.1558-5646.2012.01603.x 22759287

[59] Bonckaert W, Drijfhout FP, d’Ettorre P, Billen J, Wenseleers T. Hydrocarbon signatures of egg maternity, caste membership and reproductive status in the common wasp. J Chem Ecol. 2012;38:42–51. 10.1007/s10886-011-0055-9 22234429

