# Supplementary material for: Effects of juvenile hormone in fertility and fertility-signaling in workers of the common wasp Vespula vulgaris
Source: PLoS One. 2021 May 17;16(5):e0250720. doi: 10.1371/journal.pone.0250720 (PMC8128253; doi:10.1371/journal.pone.0250720)
Supplement: S1 Table — Retention time (min), Kovats retention indices and diagnostic ions per compound. (DOCX) [file pone.0250720.s002.docx]

S1 Table: Identification of 68 compounds in the cuticle of workers (n=308) and queens (n=16). Retention time (min), Kovats retention indices and diagnostic ions per compound.

| Retention time (min) | Retention index | Compound | Diagnostic ions |
| --- | --- | --- | --- |
| 19.52 | 2108 | C21 | 296 |
| 20.03 | 2151 | 2,12+2,14+2,16-DiMeC21 | 42 85 197 224 253 295 |
| 20.71 | 2207 | C22 | 310 |
| 21.40 | 2265 | 4-MeC22 | 71 281 |
| 21.69 | 2290 | C23:1 | 320 |
| 21.89 | 2306 | C23 | 324 |
| 22.33 | 2342 | 9+11-MeC23 | 140 168 196 224 |
| 22.39 | 2348 | 7-MeC23 | 112 253 |
| 22.51 | 2357 | 5-MeC23 | 85 281 |
| 22.77 | 2379 | 3-MeC23 | 57 309 |
| 22.90 | 2389 | 5,9-diMeC23 | 85 151 295 |
| 23.09 | 2405 | C24 | 338 |
| 23.20 | 2414 | 3,9+3,7-diMeC23 | 127 155 225 253 323 |
| 23.53 | 2440 | 9+10+11+12-MeC24 | 140 154 168 182 196 210 225 |
| 23.66 | 2451 | 6-MeC24 | 98 281 |
| 23.72 | 2456 | 4-MeC24 | 71 309 |
| 23.81 | 2463 | 3-MeC24 | 57 323 |
| 24.05 | 2482 | C25:1 | 350 |
| 24.34 | 2505 | C25 | 352 |
| 24.7 | 2540 | 11+13-MeC25 | 168 196 224 |
| 24.86 | 2547 | 7-MeC25 | 112 280 |
| 24.97 | 2556 | 5-MeC25 | 85 309 |
| 25.15 | 2570 | 9,13-diMeC25 | 121 196 211 267 |
| 25.26 | 2579 | 3-MeC25 | 57 337 |
| 25.37 | 2588 | 5,9-dimeC25 | 85 155 253 323 |
| 25.58 | 2604 | C26 | 366 |
| 25.69 | 2613 | 3,9+3,11-dimeC25 | 155 183 225 253 281 000 |
| 26.01 | 2638 | 12-MeC26 | 182 224 |
| 26.17 | 2651 | 6-MeC26 | 98 208 |
| 26.33 | 2664 | 4-Mec26 | 71 337 |
| 26.58 | 2683 | 4,12-diMeC26 | 71 225 |
| 26.74 | 2696 | C27:1 | 83 97 111 379 |
| 26.87 | 2706 | C27 | 380 |
| 27.28 | 2738 | 9+11+13-MeC27 | 196 224 280 |
| 27.39 | 2747 | 7-MeC27 | 112 309 |
| 27.51 | 2757 | 5-MeC27 | 85 337 |
| 27.64 | 2767 | 11,15-diMeC27 | 168 196 239 267 |
| 27.81 | 2780 | 3-MeC27 | 57 365 |
| 27.91 | 2788 | 5,13-diMeC27 | 85 211 224 351 |
| 28.13 | 2806 | C28 | 394 |
| 28.22 | 2813 | 3,9+3,11+3,13-diMeC27 | 57 183 211 224 253 281 379 |
| 28.43 | 2829 | 12+14+16-MeC28 | 182 210 225 253 337 365 |
| 28.89 | 2865 | 4-MeC28 | 70 366 |
| 29.15 | 2886 | C29:1 | 406 |
| 29.27 | 2895 | 4,12+4,14-diMeC28 | 70 197 225 253 380 |
| 29.41 | 2906 | C29 | 408 |
| 29.81 | 2938 | 11+13+15-MeC29 | 168 196 224 252 281 |
| 29.93 | 2948 | 7-MeC29 | 112 336 |
| 30.06 | 2958 | 5-MeC29 | 85 365 |
| 30.16 | 2966 | 11,19-DiMeC29 | 169 295 |
| 30.35 | 2981 | 3-MeC29 | 57 393 |
| 31.07 | 3039 | C30 | 422 |
| 31.27 | 3055 | 13-MeC30 | 196 267 |
| 31.41 | 3067 | 4-MeC30 | 71 393 |
| 31.69 | 3089 | diMeC30 | 169 197 225 252 281 309 366 407 436 |
| 31.78 | 3097 | C31:1 | 435 |
| 31.92 | 3108 | C31 | 436 |
| 32.30 | 3139 | 11+13+15 MeC31 | 168 196 225 252 289 309 |
| 32.68 | 3171 | 11,19-diMeC31 | 168 196 295 323 |
| 32.85 | 3184 | 3-MeC31 | 57 421 |
| 33.53 | 3241 | 2,8-diMeC31 | 42 280 211 450 |
| 34.72 | 3342 | 11+13+15-MeC33 | 168 196 308 450 463 |
| 35.08 | 3372 | C34 | 478 |
| 35.29 | 3390 | 12+14+16-MeC34 | 183 211 239 281 309 |
| 36.27 | 3475 | 6,22-DiMeC34 | 196 337 436 |
| 36.52 | 3497 | C35 | 492 |
| 37.43 | 3578 | C36 | 506 |
