# Supplementary material for: Effects of juvenile hormone in fertility and fertility-signaling in workers of the common wasp Vespula vulgaris
Source: PLoS One. 2021 May 17;16(5):e0250720. doi: 10.1371/journal.pone.0250720 (PMC8128253; doi:10.1371/journal.pone.0250720)
Supplement: S2 Table — (DOCX) [file pone.0250720.s003.docx]

S2 Table: Initial number of treated wasps and total number of treated alive and dead wasps during our experiment (NA: not available).

| colony | treatment | initial | alive | dead | nestbox |
| --- | --- | --- | --- | --- | --- |
| Vv_6_2017 | methoprene | NA | 11 | NA | A |
| Vv_6_2017 | ace-met | NA | 6 | NA | A |
| Vv_6_2017 | precocene | NA | 10 | NA | B |
| Vv_6_2017 | ace-prec | NA | 4 | NA | B |
| Vv_7_2017 | methoprene | 24 | 4 | 20 | C |
| Vv_7_2017 | ace-met | 24 | 17 | 7 | C |
| Vv_7_2017 | precocene | 24 | 16 | 8 | D |
| Vv_7_2017 | ace-prec | 24 | 17 | 7 | D |
| Vv8_2017 | methoprene | 18 | 12 | 6 | E |
| Vv8_2017 | ace-met | 18 | 8 | 10 | E |
| Vv8_2017 | precocene | 18 | 16 | 2 | F |
| Vv8_2017 | ace-prec | 18 | 12 | 6 | F |
| Vv9_2017 | methoprene | 31 | 17 | 15 | G |
| Vv9_2017 | ace-met | 31 | 25 | 6 | G |
| Vv9_2017 | precocene | 31 | 21 | 10 | H |
| Vv9_2017 | ace-prec | 31 | 14 | 17 | H |
| Vv10_2017 | methoprene | 21 | 11 | 10 | I |
| Vv10_2017 | ace-met | 21 | 17 | 4 | I |
| Vv10_2017 | precocene | 21 | 18 | 3 | J |
| Vv10_2017 | ace-prec | 21 | 13 | 8 | J |
| Vv11_2017 | methoprene | 19 | 12 | 7 | K |
| Vv11_2017 | ace-met | 19 | 12 | 7 | K |
| Vv11_2017 | precocene | 19 | 7 | 12 | L |
| Vv11_2017 | ace-prec | 19 | 15 | 4 | L |
