# Supplementary material for: Effects of juvenile hormone in fertility and fertility-signaling in workers of the common wasp Vespula vulgaris
Source: PLoS One. 2021 May 17;16(5):e0250720. doi: 10.1371/journal.pone.0250720 (PMC8128253; doi:10.1371/journal.pone.0250720)
Supplement: S3 Table — Binomial GLMM, with colony and nest box as random intercept. To check significant differences between treatments were calculated using Tukey post-hoc test (function lsmeans of the emmeans package). Odds-ratio, standard errors, z-ratio and p-values are shown. (DOCX) [file pone.0250720.s004.docx]

S3 Table: Pairwise comparison of the proportion of individuals that died in the treatments, showing that methoprene treated individuals were different from acetone control and precocene. Binomial GLMM, with colony and nest box as random intercept. To check significant differences between treatments were calculated using Tukey post-hoc test (function lsmeans of the emmeans package). Odds-ratio, standard errors, z-ratio and p-values are shown.

| **contrast** | **odds.ratio** | **SE** | **z.ratio** | **p.value** |  |
| --- | --- | --- | --- | --- | --- |
| (ace-met) / (ace-prec) | 0.74 | 0.24 | -0.91 | 0.80 |  |
| (ace-met) / methoprene | 0.42 | 0.12 | -3.11 | 0.01 | * |
| (ace-met) / precocene | 0.98 | 0.32 | -0.05 | 1.00 |  |
| (ace-prec) / methoprene | 0.56 | 0.18 | -1.83 | 0.26 |  |
| (ace-prec) / precocene | 1.32 | 0.38 | 0.99 | 0.76 |  |
| methoprene / precocene | 2.36 | 0.76 | 2.67 | 0.04 | * |
